# Association between visceral fat area and diabetic retinopathy among people with type 2 diabetes mellitus: a cross-sectional study in Ningbo, Zhejiang Province, China

**DOI:** 10.3389/fmed.2024.1327805

**Published:** 2024-02-13

**Authors:** Shanshan Hua, Dongwei Yao, Siteng Wu, Miao Chen, Li Li, Bo Li

**Affiliations:** ^1^Department of Ophthalmology, The First Affiliated Hospital of Ningbo University, Ningbo, Zhejiang, China; ^2^Department of Endocrinology and Metabolism, The First Affiliated Hospital of Ningbo University, Ningbo, Zhejiang, China

**Keywords:** visceral fat area, diabetic retinopathy, China, type 2 diabetes mellitus, central obesity

## Abstract

**Aim:**

The objective of this study is to investigate the relationship between visceral fat area (VFA) and diabetic retinopathy (DR) in the context of type 2 diabetes mellitus (T2DM) within Ningbo, China.

**Methods:**

The data of a total of 3,707 subjects with T2DM treated at The First Affiliated Hospital of Ningbo University were enrolled. The existence and severity of diabetic retinopathy were assessed by employing the 45° two-field stereoscopic digital photography. Subjects were categorized into four distinct groups: those without DR (NDR), individuals with mild non-proliferative DR (mild NPDR), people with moderate non-proliferative DR (moderate NPDR), and those suffering from vision-threatening DR (VTDR). Bio-electrical impedance was employed to estimate the Visceral fat area (VFA). Multinomial logistic regression models were utilized to evaluate the association between VFA and DR.

**Results:**

The mean VFA in patients without diabetic retinopathy (NDR) was notably lower compared to that of patients with diabetic retinopathy (DR) (85.21 ± 37.78 vs. 97.37 ± 44.58 cm^2^, *p* < 0.001). As the severity of DR increased, VFA increased gradually but insignificantly (94.41 ± 43.13 cm^2^, 96.75 ± 40.82 cm^2^, 100.84 ± 49.34 cm^2^, *p* = 0.294). After adjusting the confounding factors, there was an association identified between VFA and the occurrence of DR (OR = 1.020, 95% CI = 1.016–1.024). It showed that regardless of BMI, whether it’s less than 25 kg/m^2^ or greater than or equal to 25 kg/m^2^, a higher VFA (≥100 cm^2^) level came with a higher prevalence of DR (*p* < 0.001).

**Conclusion:**

The outcomes of this research indicate a modest association between VFA and the incidence of DR among Chinese patients who have been diagnosed with T2DM in Ningbo.

## 1 Introduction

It’s forecasted that the worldwide prevalence of T2DM will surge dramatically in the upcoming decades, marking it a major concern for global public health. By 2030, it’s projected that around 643 million individuals will be diagnosed with diabetes (1). Research has demonstrated that approximately 50% of individuals with T2DM experience microvascular complications (2). Diabetic retinopathy, as the most prevalent microangiopathic consequence of diabetes, stands as the primary cause of sight deterioration and global blindness among middle-aged adults in the workforce (3). The duration of diabetes and hyperglycemia are widely recognized as key elements in the onset and advancement of diabetic retinopathy. The risk factors associated with DR remain inadequately elucidated. Hence, pinpointing changeable factors for DR is crucial in preventing the disease from becoming a serious health issue among the public.

In epidemiological studies, body mass index (BMI) is commonly used as a measure of overall obesity, indicating an approximate overall body fat percentage. Whereas, metrics such as VFA, waist-hip ratio (WHR), or waist circumference (WC) are utilized to quantify abdominal or visceral obesity, providing a reflection of regional fat accumulation (4). There may be some ethnic disparity as Asians generally exhibit a higher predisposition toward abdominal obesity accumulation compared to White people (5, 6). While WC or WHR are typically used as indicators for detecting abdominal obesity, they fall short in differentiating the mass of subcutaneous fat from that of visceral fat, despite these two types of fat having distinct functions. Conversely, VFA remains unaffected by the confounding influence of subcutaneous fat, hence making it a more accurate and reliable measure than WC or WHR. Furthermore, for the Chinese population, measures of visceral adiposity such as VFA have gained attention as superior risk predictors compared to generalized obesity metrics like BMI. Also referred to as central obesity, abdominal obesity is prevalent in patients struggling with T2DM. It is primarily distinguished by an excessive buildup of visceral fat in the abdominal region. Its role as a risk factor for the development of T2DM is indisputably established (7, 8). Numerous studies have been conducted to exploring the correlation between the abdominal obesity and DR. However, previous studies are inconsistent in results. While some research has suggested that abdominal obesity could result in a heightened risk of DR, other studies have reported either negligible correlation or a significant reverse relationship between abdominal obesity and DR (9–13). Furthermore, in China, only a handful of studies have delved into the correlation between abdominal obesity, as determined by VFA, and the risk of DR.

Hence, the main objective of our study was to observe the variations in VFA across various stages of DR, and to investigate the link between abdominal obesity and both the existence and severity of DR among T2DM patients residing in Ningbo, China.

## 2 Materials and methods

### 2.1 Study participants

We performed a cross-sectional study, gathering pertinent data from the National Metabolic Management Center (MMC) at The First Affiliated Hospital of Ningbo University over a period spanning from April 2019 to April 2022.MMC is a program based in multiple hospitals across mainland China led by the Shanghai-based Ruijin Hospital for providing standardized management of metabolic diseases (14). The criteria for inclusion in this study were as follows: (1) T2DM; (2) gradable fundus examination; (3) availability of VFA data; and (4) age > 18. The determination of T2DM in the subjects was confirmed following the 1999 World Health Organization’s diagnostic criteria for diabetes mellitus (15). The criteria for exclusion in this study were as follows: (1) pregnancy; (2) acute complications related to diabetes like diabetic hyperosmolar coma, diabetic ketoacidosis; or (3) a track record of severe systemic diseases apart from diabetes, including serious cerebrovascular/ cardiovascular diseases and malignant tumors. A total of 336 participants were excluded from this study (including 14 patients under 18 years of age, 98 patients with missing visceral fat area data and 224 patients with missing fundus imaging data). As a result, 3,707 participants were ultimately incorporated in the analysis.

The Research Ethics Committee of The First Affiliated Hospital of Ningbo University granted ethical approval for the study (2019-R057), adhering to the guidelines laid out in the Declaration of Helsinki. Furthermore, every participant provided written informed consent.

### 2.2 Clinical examination and interview

Trained nurses and laboratory staff measured and analyzed the physiological, anthropometric, and biochemical parameters using a standardized protocol by MMC. The patients’ fundamental information and anthropometric features were assessed based on the following parameters: gender, age, body weight, body mass index (BMI), disease duration, height and visceral fat area.

### 2.3 Laboratory techniques

Blood samples were collected from the patient’s antecubital vein in the morning following an overnight fast. All patients were subjected to standard laboratory examinations using conventional biochemical analysis techniques. These included serum triglyceride (TG), serum total cholesterol (TC), glycated hemoglobin (HbA1c), fasting plasma glucose (FPG), low density lipoprotein cholesterol (LDL-c),high density lipoprotein cholesterol (HDL-c), uric acid (UA), creatinine and blood urea nitrogen (BUN). The evaluation of urinary albumin was carried out using spot urine samples via a solid-phase competitive chemiluminescent enzymatic immunoassay, and the albumin-to-creatinine ratio (ACR) in urine was determined. The level of HbA1c was gauged by high-performance liquid chromatography. Lipids were measured with enzymatic methods.

### 2.4 Assessment of VFA

VFA and subcutaneous fat were calculated at the umbilical level using a dual bio-electrical impedance method (DUALSCAN HDS-2000, Omron, Japan). Measurement required patients to maintain a fasting period of at least 8 h beforehand. The method involved the following steps: (1) Instructions were given to subjects to lie flat on their backs, reveal their ankles, wrists, and abdomen, maintain a calm breathing pattern, and cease their breathing at the end of a relaxed exhalation. At this step, the horizontal umbilical abdominal cross-sectional area was measured; (2) The electrode belt was placed around the subjects’ abdomen and electrode clips were applied to their hands and feet. Following these preparations, subjects were asked to maintain a calm and steady breath. They were then informed to suspend their breathing at the end of a peaceful exhalation. The instrument automatically calculated the abdominal visceral fat area, which was recorded in cm^2^. VFA levels were categorized into two groups: those equal to or exceeding 100 cm^2^ were classified as high VFA, while those falling below 100 cm2 were deemed as normal VFA.

### 2.5 Fundus photography and evaluation of DR

A trained photographer took two-field digital color images of the fundus from each eye of all participants. These images were focused on the macula’s focal point and the optic disc without mydriasis condition. In accordance with the International Classification of diabetic retinopathy, a retinal specialist analyzed each image to ascertain the presence and assess the severity of DR (16). The result input and verification are carried out by two experts to avoid input errors. Participants with no abnormalities observed in fundus photographs were classified as without diabetic retinopathy (NDR, *n* = 3,010). On the other hand, participants displaying symptoms such as microaneurysms, hemorrhages, hard exudates, venous beading, intraretinal microvascular abnormalities, cotton wool spots, preretinal new vessels, fibrous proliferation, and scars of photocoagulation were identified as those suffering from diabetic retinopathy (DR). According to severity, diabetic retinopathy (DR) was categorized into mild nonproliferative diabetic retinopathy (NPDR, *n* = 224), moderate NPDR (*n* = 240), severe NPDR, and proliferative diabetic retinopathy (PDR). Given the complexity of distinguishing between severe NPDR and PDR solely from fundus images, severe NPDR and PDR were grouped together as vision-threatening diabetic retinopathy (VTDR, *n* = 233) (17, 18). Data from the worse eye were adopted.

### 2.6 Statistical analysis

The bulk of statistical analyses were carried out utilizing the SPSS 22.0 statistical software package (IBM Corp., NY, United States). Continuous variables were reported as either mean ± standard deviation (SD) or median with interquartile range, while categorical data was represented as the number of occurrences (N) and the corresponding percentages (%). The participant characteristics, divided into those with DR and those without, were compared by employing the Chi-square test for proportion comparisons. Meanwhile, the t-test or Mann–Whitney U test was employed for comparing means or medians, as suited to the distribution of the data. Either a One-way ANOVA or Kruskal-Wallis H test was employed to assess the differences of continuous variables across mild, moderate, and VTDR categories. The associations between VFA and other variables were examined using either Spearman or Pearson correlation analysis. The impact of VFA on DR was determined using logistic regression analysis across four distinct models. Model 1 involved no adjustments; Model 2 included adjustments for gender, age and the duration of T2DM; Model 3 added further adjustments in HbA1c, weight, BMI, TC, TG, LDL and HDL, and model 4 was further adjusted in UA, Cr, BUN, ACR, urine microalbumin and urine creatinine, respectively. Values of regression coefficients (β) and their respective 95% confidence intervals (CIs) were documented. Any value of *p* ≤0.05 was deemed to indicate statistical significance.

## 3 Results

Table 1 presents the clinical and demographic attributes of the participants involved in the study. Out of the 3,707 qualifying participants, a majority of 3,010 (81.2%) displayed no indicators of DR, while 697 (18.8%) were diagnosed with DR. As depicted in Table 1, a significant disparity was noted in the VFA between the DR group and the NDR group. The DR group exhibited a notably higher VFA level compared to that of the NDR group (97.37 ± 44.58 vs. 85.21 ± 37.78, *p* < 0.001). In relation to those without DR, participants who had DR were elder, had lower BMI, lower subcutaneous fat area, longer diabetes duration, higher HbA1c levels, higher fasting plasma glucose, lower LDL-c, lower TC, higher TG, higher Cr, higher BUN, higher ACR, as well as higher urine microalbumin and urine creatinine levels (all *p* < 0.05).

**Table 1:** Clinical and demographic characteristics of 3,707 participants classified by the presence of DR.

Characteristics	NDR *N* = 3,010 (81.2%)	DR *N* = 697 (18.8%)	*p*-value
Men (%)	64.4%	62.1%	0.262
Age (y)	50.40 ± 11.84	56.59 ± 10.62	< 0.001
Weight (kg)	70.12 ± 13.04	67.27 ± 11.95	<0.001
BMI (kg/m^2^)	25.55 ± 3.68	25.04 ± 3.53	0.001
VFA (cm^2^)	85.21 ± 37.78	97.37 ± 44.58	< 0.001
Subcutaneous fat area (cm^2^)	181.38 ± 61.57	171.0 ± 58.86	< 0.001
Diabetes duration (years)	4.92, (1.67,10)	10, (5,15.58)	<0.001
HbA1_c_ (%)	6.10 ± 2.01	9.05 ± 2.62	<0.001
FPG (mmol/L)	8.75 ± 2.97	9.27 ± 3.70	0.001
LDL-c (mmol/L)	3.18 ± 0.90	3.03 ± 0.95	<0.001
HDL-c (mmol/L)	1.18 ± 0.28	1.18 ± 0.31	0.896
TC (mmol/L)	4.85 ± 1.20	4.72 ± 1.36	0.016
TG (mmol/L)	1.20 (0.74,1.93)	1.47 (1.06,2.14)	<0.001
UA (umol/L)	334.74 ± 86.76	340.48 ± 92.38	0.142
Cr (umol/L)	63.65 ± 17.96	69.50 ± 34.96	<0.001
BUN (mmol/L)	5.23 ± 1.46	6.03 ± 2.20	<0.001
ACR	10.9 (6.06, 27.0)	21.256 (8.43,90.74)	<0.001
Urine microalbumin (mg/L)	14.9 (7.2,34.67)	21.45 (8.86, 94.89)	<0.001
Urine creatinine (mg/dL)	10816.5 (6713.5,16186.25)	8289.5 (5,133, 12445.25)	<0.001

NDR, no diabetic retinopathy; DR, diabetic retinopathy; BMI, body mass index; VFA, visceral fat area; HbA1_c_, glycated hemoglobin; FPG, fasting plasma glucose; LDL-c, low density lipoprotein-cholesterol; HDL-c, high density lipoprotein- cholesterol; TC, total cholesterol; TG, triglycerides; UA, Uric Acid; Cr, creatinine; BUN, Blood urea nitrogen; ACR, urinary albumin: creatinine ratio.

Among subjects diagnosed with T2DM, there exists a link between the VFA and other different variables, which is detailed in Table 2. A statistically meaningful positive correlation was identified between VFA and several health indicators. These indicators include DR (*r* = 0.111, *p* < 0.001), HbA1C (*r* = 0.067, *p* < 0.001), TC (*r* = 0.70, *p* < 0.001), TG (*r* = 0.394, *p* < 0.001), LDL (*r* = 0.079, *p* < 0.001), Cr (*r* = 0.152, *p* < 0.001), BUN (*r* = 0.044, *p* = 0.009) and ACR (*r* = 0.200, *p* < 0.001). Conversely, there was a statistically significant negative correlation between HDL (*r* = −0.255, *p* < 0.001) and VFA.

**Table 2:** Association between VFA and other variables.

Variable	*r*	*p*
Age (years)	0.23	0.160
HbA1c (%)	0.067	< 0.001
Duration of diabetes (years)	0.003	0.861
TC (mmol/L)	0.70	< 0.001
TG (mmol/L)	0.394	< 0.001
HDL-c (mmol/L)	−0.255	< 0.001
LDL-c (mmol/L)	0.079	< 0.001
Cr (μmol/L)	0.152	< 0.001
BUN (mmol/L)	0.044	0.009
ACR	0.200	< 0.001
DR	0.111	< 0.001

VFA, visceral fat area; HbA1c, glycated hemoglobin; TC, total cholesterol; TG, triglycerides; HDL-c, high density lipoprotein cholesterol; LDL-c, low-density lipoprotein-cholesterol; Cr, creatinine; BUN, blood urea nitrogen; ACR, urinary albumin: creatinine ratio; DR, diabetic retinopathy.

### 3.1 Associations of VFA with the occurrence of DR

Table 3 presents a logistic regression model highlighting the association between diabetic retinopathy and VFA. From Model 1 of univariable logistic regression, we discovered a significant association between VFA (OR: 1.007; 95% CI: 1.005–1.010; *p* < 0.001) and the occurrence of DR. After adjusting of different confounding factors (gender, age, and T2DM duration) in Model 2, we observed a continued association of higher VFA with the incidence of DR (OR: 1.009; 95% CI: 1.006–1.011; *p* < 0.001). This correlation continued to be significant even after adjusting for HbA1c, weight, BMI, TC, TG, LDL, and HDL (Model 3 in Table 3; OR: 1.019, 95% CI: 1.015–1.023, *p* < 0.001). Furthermore, upon additional adjustment for other confounders (UA, Cr, BUN, ACR, urine microalbumin and urine creatinine) in Model 4, a significant association still persisted between VFA (OR = 1.020, 95% CI = 1.016–1.024) and the occurrence of DR (*p* < 0.001).

**Table 3:** Logistic regression analysis of VFA and DR.

Variable	Model 1		Model 2		Model 3		Model 4	
	OR (95% CI)	*p*-value	OR (95% CI)	*p-*value	OR (95% CI)	*p*-value	OR (95% CI)	*p*-value
VFA	1.007, (1.005,1.010)	<0.001	1.009, (1.006,1.011)	<0.001	1.019, (1.015,1.023)	<0.001	1.020, (1.016,1.024)	<0.001

Model 1: unadjusted. Model 2: Model 1 + adjusted gender, age, and T2DM duration. Model 3: Model 2 + adjusted HbA1c, weight, BMI, TC, TG, LDL and HDL. Model 4: Model 3 + adjusted UA, Cr, BUN, ACR, urine microalbumin and urine creatinine. VFA, visceral fat area; DR, diabetic retinopathy; T2DM, type 2 diabetes mellitus; HbA1c, glycated hemoglobin; BMI, body mass index; TC, total cholesterol; TG, triglycerides; HDL, high density lipoprotein; LDL, low density lipoprotein; UA, Uric Acid; Cr, creatinine; BUN, blood urea nitrogen; ACR, urinary albumin: creatinine ratio.

**Figure 1:**
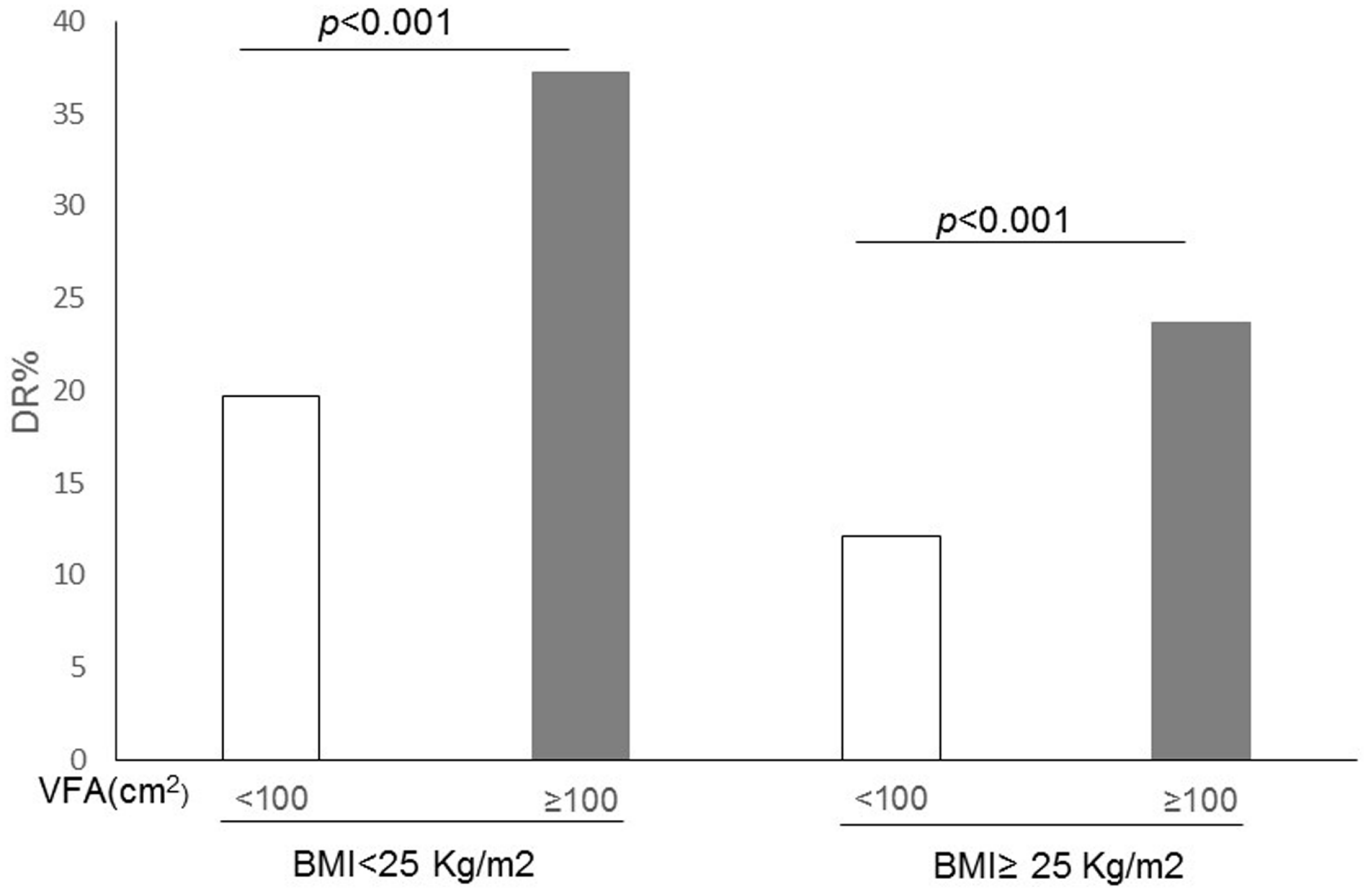
Correlation between the incidence of diabetic retinopathy (DR) and both visceral fat area (VFA) and body mass index (BMI) in patients with type 2 diabetes.

The levels of VFA were categorized into two groups: those with levels equal to or greater than 100 cm^2^ were classified as having high VFA, while those falling below 100 cm^2^ were deemed as normal VFA. It showed that regardless of BMI (<25 kg/m^2^ or ≥ 25 kg/m^2^), patients with high VFA levels had more retinopathy than those of normal VFA levels (*p* < 0.001) (Figure 1).

### 3.2 Associations of VFA with the severity of DR

Table 4 displays the clinical and demographic characteristics of the 697 study participants, categorized by the severity of their retinopathy. As indicated in Table 4, there is a modest yet statistically insignificant increase in VFA values as the severity of DR escalates (94.41 ± 43.13 cm^2^, 96.75 ± 40.82 cm^2^, 100.84 ± 49.34 cm^2^, *p* = 0.294).

**Table 4:** Clinical and demographic characteristics of 697 participants according to the severity of retinopathy.

Parameter	Mild DR *N* = 224	Moderate DR *N* = 240	Severe DR *N* = 233	*p*
Men%	61.2%	63.3%	61.8%	0.883
Age (y)	55.39 ± 10.51	55.18 ± 10.34	59.19 ± 10.58	< 0.001
Weight (kg)	68.55 ± 11.78	67.13 ± 11.64	66.18 ± 12.36	0.104
BMI (kg/m^2^)	25.44 ± 3.63	24.83 ± 3.24	24.86 ± 3.70	0.116
VFA (cm^2^)	94.41 ± 43.13	96.75 ± 40.82	100.84 ± 49.34	0.294
Subcutaneous fat area (cm^2^)	177.22 ± 59.21	168.12 ± 57.80	167.98 ± 59.39	0.158
Diabetes duration (years)	9.58, (4.81, 15)	10, (4.58, 15.58)	10.83, (6, 17.08)	0.014
HbA1_c_%	7.37 ± 2.12	8.80 ± 2.07	10.91 ± 2.37	< 0.001
FPG (mmol/L)	9.16 ± 3.36	9.31 ± 3.80	9.34 ± 3.91	0.861
LDL-c (mmol/L)	3.02 ± 0.96	3.00 ± 0.89	3.06 ± 1.01	0.790
HDL-c (mmol/L)	1.17 ± 0.29	1.18 ± 0.33	1.18 ± 0.29	0.924
TC (mmol/L)	4.66 ± 1.27	4.71 ± 1.37	4.78 ± 1.43	0.673
TG (mmol/L)	1.47 (1.045, 2.11)	1.33 (0.96, 2.17)	1.64, (1.2, 2.18)	0.004
UA (umol/L)	337.20 ± 90.57	337.69 ± 91.41	346.56 ± 95.17	0.482
Cr (umol/L)	65.49 ± 18.33	68.42 ± 31.26	74.51 ± 47.99	0.020
BUN (mmol/L)	5.68 ± 1.90	5.96 ± 2.19	6.42 ± 2.42	0.002
ACR	13.61 (6.67, 39.03)	18.62 (7.33, 83.3)	39.98 (12.91, 238.26)	<0.001
Urine microalbumin (mg/L)	16.5 (7.33, 46.75)	17.8 (7.98, 83.34)	45.7 (13.22, 211.78)	<0.001
Urine creatinine (mg/dL)	8,842 (5,400, 12,942)	8,316 (5,363, 12,420.5)	7,415 (4,773.3, 11,792.78)	0.055

DR, diabetic retinopathy; BMI, body mass index; VFA, visceral fat area; HbA1c, glycated hemoglobin; FPG, fasting plasma glucose; LDL-c, low-density lipoprotein-cholesterol; HDL-c, high density lipoprotein- cholesterol; TC, total cholesterol; TG, triglycerides; UA, Uric Acid; Cr, creatinine; BUN, Blood urea nitrogen; ACR, urinary albumin: creatinine ratio.

## 4 Discussion

Our findings indicate a significant correlation between VFA, an indicator of abdominal obesity, and the risk of DR. Even after accounting for potential confounding factors, this association remained consistent, suggesting that the accumulation of abdominal fat may indeed have a genuine impact on the development of DR. Therefore, VFA could potentially serve as a helpful marker for pinpointing T2DM patients at risk of DR. It’s hypothesized that the distribution of visceral fat might have an impact on the development of DR. However, in our study, we found no considerable statistical correlation between abdominal obesity and the various stages of severity in DR.

### 4.1 Relationship between abdominal obesity and DR

Various studies have assessed the correlation between abdominal obesity and DR, yet previous studies have been inconsistent in results. In their research, Wu and colleagues (11) discovered a correlation between the index of abdominal obesity and diabetic nephropathy, but had no significant correlation with DR in the Chinese. Nonetheless, they also clarified that in this cohort study, the cumulative prevalence of diabetic retinopathy was merely 1.0%, indicating that the participants were primarily in the relatively initial phases of diabetes. In addition, according to the annual physical exams of the participants, their blood glucose was well-controlled, and even their glycosylated hemoglobin were not of high levels. A study in 2020 also found that Chinese visceral adiposity index was significantly correlated with cerebrovascular disease and diabetic nephropathy. However, this association was not found with diabetic retinopathy in either gender (19). In the study, a comprehensive count of 179 subjects were incorporated. This included 110 patients suffering from diabetes but without any signs of DR, and another group of 69 individuals who, while being diabetic, exhibited symptoms of DR. MRI was used to measure the distribution of fat. The average area of visceral fat among all groups was far greater than 200 cm^2^, and it was concluded that the distribution of visceral and subcutaneous fat was not related to diabetic retinopathy (13). Iwasaki et al. (20) found that in Japanese patients with T2DM, an escalation in waist circumference (WC) did not increase the risk of DR. Additionally, a community-sourced cross-sectional examination which involved 1,184 Indonesian individuals with T2DM showed that the measurements of body composition demonstrated an inverse correlation to both the occurrence and severity of DR (21). A study on Korean men showed a negative correlation between abdominal obesity and vision-threatening DR. This could potentially be ascribed to the unintended weight reduction owing to ill-managed blood glucose levels and harsh diabetic conditions (12).

Nonetheless, the results of many other studies are consistent with our data. The SN-DREAMS-I Study, for example, revealed that in urban South India, specific abdominal obesity and heightened Waist to Hip Ratio in females were correlated with the presence of diabetic retinopathy among Indians. However, these factors did not show a correlation with the severity of the condition (22). Moh et al. (9) determined a linkage between elevated visceral adiposity and DR in Asian subjects with long-standing T2DM. This observation suggests that reduction of visceral fat could potentially slow down the development of DR, even in cases of a long duration of diabetes. Visceral adiposity could potentially have a connection to vascular damage, thereby playing a significant role in the underlying pathophysiology of DR. Anan et al. (10) illustrated a correlation between the increase in VFA and DR, but interestingly, in the Japanese population with T2DM, BMI and WC did not serve as standalone risk factors for DR. The Hoorn study in the Netherlands also concluded that abdominal obesity was an independent variable for retinopathy (23). The study from the Singapore Diabetes Management Project indicated that abdominal obesity (determined by WHR) showed a positive correlation with the existence of any degree of DR. Interestingly, the correlation between WHR and the severity of DR was detected exclusively in women (24). Han’s recent study provided longitudinal evidence that abdominal obesity amplified the risk of developing DR, yet it failed to show a significant correlation with the 2-year progression of DR in Chinese patients. This aligns with the observations from our research (25). The results of a meta-analysis showed an association between abdominal obesity, as quantified by Waist Circumference and Waist-Hip Ratio, and DR. Nevertheless, the study found no association between the varying severities of DR and abdominal obesity (26). Taken together, we believe that the variations in data obtained from different researches could be partly attributed to discrepancies in elements such as study design, gender of subjects, ethnicity, levels of obesity, severity of DR, and detection methods.

### 4.2 Mechanisms between abdominal obesity and DR

Currently, although the pathophysiological dynamics substantiating the link between abdominal obesity and DR are not entirely understood, a number of prospective hypotheses have been put forward. The excessive build-up of visceral fat in the abdominal area could potentially instigate an elevated release of free fatty acids. Consequently, this could pave the way for insulin resistance and inflammation within adipose tissues (27–29). Potential consequences of insulin resistance may include the onset of hypo-inflammation (30), endothelial dysfunction (31), and an increase in oxidative stress (32). These conditions are suspected to be significant contributors to the development of DR. Research has also identified that chronic inflammation could potentially lead to the occlusion of retinal capillaries, which will further induce retinal ischemia and retinopathy (33). A recent study has found the levels of adipokines in the group diagnosed with diabetic retinopathy were surprisingly elevated as compared to the group without DR, by measuring the adipokines in aqueous humor directly. Furthermore, the study showed that the adipokine concentration was positively correlated with the abdominal obesity index. These findings propose that abdominal obesity plays a significant role in the progression of diabetic retinopathy (34). These mechanisms could partially justify the positive correlation found between VFA and the onset of DR. Nevertheless, these underlying theories of how abdominal obesity operates still necessitate further comprehensive clinical and base-level validation.

### 4.3 The advantage of VFA measured by BIA

In Asians, a visceral fat area of 100 cm^2^ or more is considered as the benchmark for diagnosing visceral obesity (35). Currently, magnetic resonance imaging (MRI) and abdominal computed tomography (CT) are regarded as the most reliable methods for quantitatively assessing the VFA (36). However, due to their time-consuming nature, risks of radiation exposure, and high costs, these methodologies are impractical for customary clinical application and widespread population-based epidemiological research. Currently, the dual bio-electrical impedance analysis (BIA) has been developed as an effective tool for measuring visceral fat, which gauges body composition by utilizing the differences in electrical currents flowing through various mediums (37). Studies have evaluated that the BIA method for estimating VFA strongly correlates with the results obtained from CT measurements (38). Meanwhile, the BIA method has the advantages of high safety, low cost, fast and simple operation, and can be widely used in clinical practices (39). Therefore, in this study, the dual bio-electrical impedance analysis method was utilized to estimate the visceral fat area. Regardless of BMI (< 25 kg/m^2^ or ≥ 25 kg/m^2^), people with high VFA levels showed more retinopathy than those with normal VFA levels (*p* < 0.001).

### 4.4 Shortcomings and limitations

This study also carries a few limitations. Primarily, its cross-sectional design of the study makes it inapplicable for cause-effect relationships. Secondly, the evaluation for DR was predicated on two-field retinal photographs instead of seven-field retinal photographs. This choice in methodology could potentially underestimate the actual occurrence of DR. Thirdly, since this study mainly targeted the people in Ningbo, Zhejiang Province, China, selection bias of patients might exist. To sum up, since obesity and DR are complex and affected by multiple factors, there is a strong need for prospective studies, with larger sample sizes and carried out in various regions, to further clarify their interconnectedness. It remains essential to conduct dynamic monitoring of the visceral fat area in order to better evaluate and predict its role on the progress of diseases.

## 5 Conclusion

Overall, this research offers persuasive evidence establishing a correlation between abdominal obesity and DR. This study’s findings imply that an elevated VFA, which is indicative of abdominal obesity, correlates to an increased risk of DR among our participants with T2DM in Ningbo, Zhejiang Province, China. However, this correlation did not extend to the severity of diabetic retinopathy. Indeed, our research results could potentially offer new perspectives that could enhance the early clinical identification, prevention, and management for diabetic retinopathy.

## Data availability statement

The original contributions presented in the study are included in the article/supplementary material, further inquiries can be directed to the corresponding authors.

## Ethics statement

The studies involving humans were approved by the Research Ethics Committee of The First Affiliated Hospital of Ningbo University (2019-R057). The studies were conducted in accordance with the local legislation and institutional requirements. The participants provided their written informed consent to participate in this study.

## Author contributions

SH: Formal analysis, Writing – original draft, Project administration, Validation, Writing – review & editing. DY: Conceptualization, Investigation, Software, Writing – original draft. SW: Methodology, Supervision, Writing – original draft. MC: Data curation, Writing – original draft. LL: Writing – review & editing, Funding acquisition, Resources. BL: Funding acquisition, Writing – review & editing.
